# Permeability enhancement of deep hole pre-splitting blasting in the low permeability coal seam of the Nanting coal mine

**DOI:** 10.1371/journal.pone.0199835

**Published:** 2018-06-28

**Authors:** Zhengyi Ti, Feng Zhang, Jin Pan, Xiaofei Ma, Zheng Shang

**Affiliations:** 1 College of Mining Engineering, Liaoning Technical University, Fu Xin, Liao Ning, China; 2 National Engineering Research Center of Coal Mine Gas Control, China University of Mining and Technology, Xu Zhou, Jiang Su, China; China University of Mining and Technology, CHINA

## Abstract

To solve the hidden danger of high methane and low permeability gas in the coal mining process, potentially affecting the safety production in an orderly way, we propose the use of deep hole blasting technology to improve the permeability of the coal seam gas drainage, increase the quantity and rate of extraction, and reduce methane output. Taking the geological conditions of the 201 working surface of Tingnan Coal Mine as an example, it is calculated that the single drilled fracture crack extension range is 3.11~5.24 m according to the coal seam deep-hole pre-splitting blasting joint mechanism and fracture propagation mechanics model, providing a theoretical basis for choosing the appropriate hole spacing. Using COMSOL simulation software to simulate the effective gas drainage radius of a coal seam from a two-dimensional perspective on a single borehole radial, the least squares fitting method was used to analyze the simulated data, and obtained the effective drilling extraction radius after pre-split blasting in a deep hole that is 3.6 m, which is in accordance with the theoretical calculations. In order to obtain accurate and scientific calculations, Fast lagrangian analysis of continua (FLAC3D) numerical simulation software was used. After simulating the distribution of plastic zone between two blast holes at different intervals from a three-dimensional angle, and evaluating the development of cracks in the blasting hole, the white zone of the blasting space was completely eliminated when the interval between blasting holes was 7 m, and the cracks could be propagated throughout the surroundings. Therefore, a blasting hole spacing of 7 m is optimal. On-site monitoring in the Nanting coal mine showed that the quantity and rate of extraction of the single hole after pre-splitting blasting were 2.36 times and 1.62 times as much as before. By integrating the borehole drainage amount and the optimized calculation equation, it could be concluded that the permeability coefficient of the coal seam after blasting was 7.78 times as much as before. The function of time-variated drilling methane emission was obtained using multivariate statistical regressions based on the on-site monitored borehole methane emission (*q*_*t*_), and the drilling limit after pre-splitting blasting revealed that the limitation of methane extraction volume was 5.27 times as much as before.

## 1 Introduction

The Nanting coal mine is a high methane mine with a methane content of 7 m^3^/t in coal seam. The absolute methane gushing quantity is 92.86 m^3^/min. The measured permeability coefficient of the coal seam is 0.25~0.69 m^2^/(MPa^2^·d) in site (poor permeability), indicating extraction difficulties. The experience of the adjacent working surfaces showed that the existence of methane overrun phenomena often significantly affected mine production. Conventional pre-pumping method was not able to solve these problems. At present, the deep hole blasting method is mainly used in the process of local anti-technology measures with the outburst coal mine. However, studies of this technology as an enhanced methane drainage method to improve extraction effects are still rare. Nilson R H [1,2] proposed the expression of the integral equation for crack propagation under fluid/gas expulsion and demonstrated the effect of the quasi-static effect of the exploding gas on crack propagation. Badal R [3] proved that the crushing of the rock mass under controlled blasting is the result of the combination of the dynamic impact shock of the blast wave and the quasi-static interaction of the exploding gas through indoor model tests. Paine A S [4] established a cylindrical blasthole model containing radial cracks, including blasthole gas evolution, ground stress, and other factors, giving the model theoretical expression and solution method. Roy P P [5] studied the innovative blasting technology used in Indian coal mines and studied drilling, explosion parameters, and gas hazards. Sang HC [6] proposed a dynamic finite element method for the interaction of stress wave, exploding gas, and cracks, and pointed out that the flow of the exploding gas in the fracture is constrained by the correlation equation of the one-dimensional transient fluid. The expansion of the rock fracture is the combination of the completed explosion wave and explosive gas. Mohammadi S [7,8] proposed the finite element-discrete element coupling method of adaptive mesh to simulate the expansion of blasting cracks in rock mass. This method can simulate the propagation process of the explosion gas in the fracture and the complex fluid-solid coupling phenomenon of the fracture interaction. Huang and Liang et al. [9,10] performed three-dimensional investigations on the mechanisms of pre-crack blasting fracture expansion and obtained the effective drilling extraction radius after blasting. Cai et al. [11,12] performed two-dimensional studies on the effect of reasonable hole spacing on the crack expansion and extraction efficiency. Currently, most researchers [13,14] focus on the extended range of pre-splitting bursts, while the report on coal seam permeability coefficient and drilling limit methane extraction is still missing, especially from both two- and three-dimensions based on the comprehensive analysis of the crack expansion range and the field test results.

In this paper, a 2D analysis of effective drilling drainage radius before and after blasting under the same vacuum conditions was simulated using COMSOL. On this basis, 3D investigation of crack propagation after blasting was carried out using FLAC3D. The optimal hole spacing of blasting drilling was obtained based on the simulated results using the least squares method. The parameters of single hole extraction, extraction rate, permeability coefficient, and drilling limit methane extraction were selected to evaluate the methane extraction. The permeability coefficient of the coal seam was calculated by the optimized equation, as the evaluation index to evaluate the extraction effect. The results of drilling limit methane extraction were calculated by multivariate statistical analysis method.

## 2 The basic principles of crack extension

**Fig 1:**
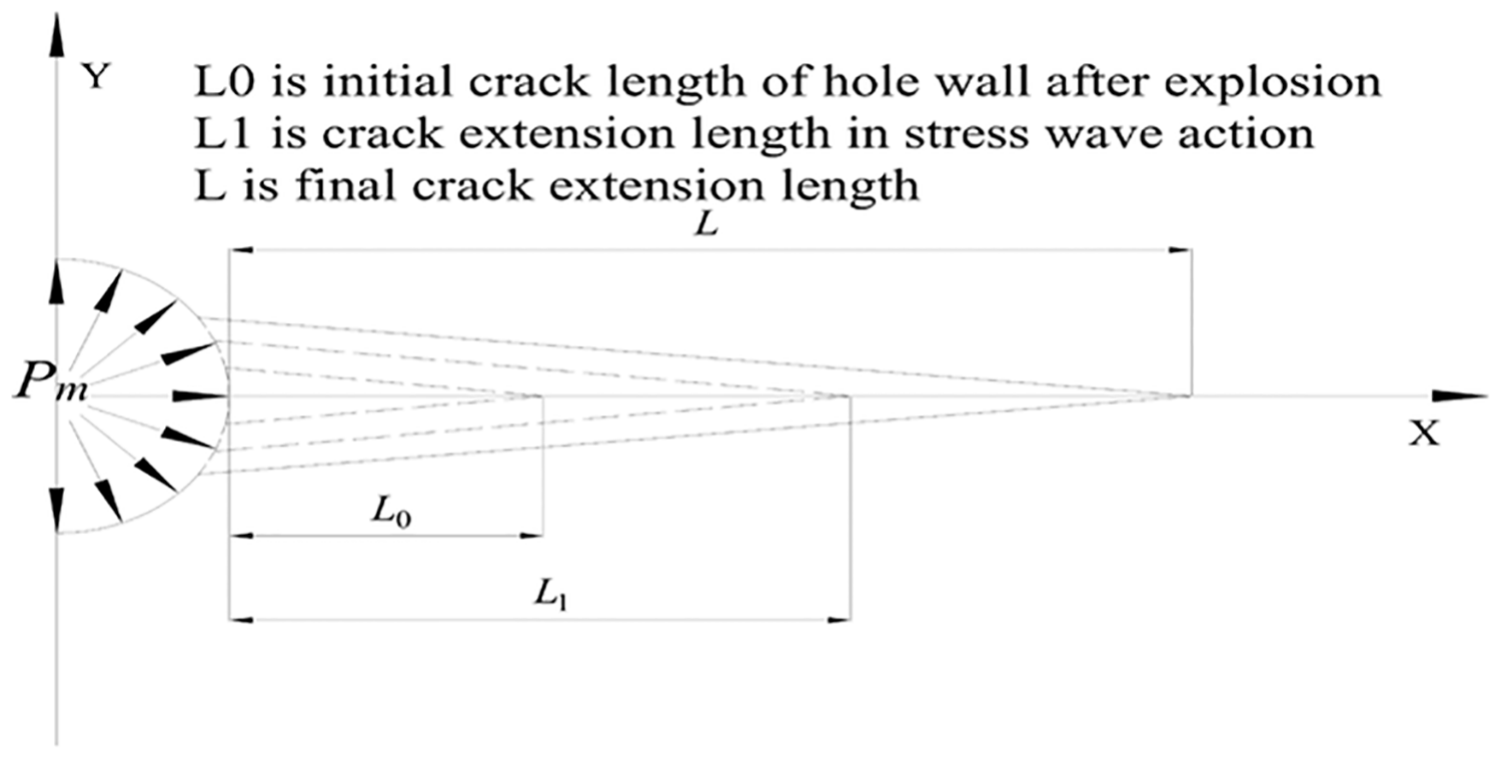
Mechanical model of crack extension.

Deep hole pre-splitting blasting aims to expand the fissure around the borehole, improve the permeability of the coal seam, and reduce the resistance in the process of extraction, which would reduce the methane content in the coal seam and prevent any potential danger in the production of methane. The extended fracture is based on the theories of equal energy and the minimum resistance line. The peak value of detonation pressure is reduced by using the discontinuous coupling charge method where σ_tensile_ is far less than σ_compressive_, creating no obvious damage caused by compression occurrences around the hole wall. The tangential tensile stress around the borehole produces a radial crack, and, due to the large amount of energy between the hole and the hole wall, the high temperature and high pressure methane is transferred outward along the crack around the hole. This forces the initial crack around the hole wall to continue to expand, causing the cracks on the surrounding line to all become connected [15,16]. Shown is the mechanical model of crack extension (Fig 1).

The pressure *P*_m_ when the initial blasting peak pressure appears after using uncoupling charge blasting process:

$$P_{m}=\frac{1}{8}\rho_{0}\cdot D^{2}\cdot \frac{1}{K^{6}}\cdot n$$ (1)

where *ρ*_0_ is the density of the explosion, kg/m^3^; *D* is the velocity of the explosion, m/s; *K* is the uncoupling coefficient; and *n* is the pressure increase constant.

The shock wave produced by the explosion would create a crushing area with a certain width around the blasting hole, followed by rapid weakening of the shock wave. The variation of peak pressure along the expanding direction could be written as:

$$P(l)=P_{m}\cdot {\left(\frac{l}{r_{b}}\right)}^{-\alpha }$$ (2)

where *l* is the distance away from the center of the blasting hole, m; *r*_b_ is the radius of the blast-holes, m; and α is the attenuation coefficient.

The internal energy of shock waves is consumed in the medium as it travels. According to the law of energy conservation, the shock wave would gradually decay into a stress wave (wave velocity is *c*_*p*_), where the peak pressure *P*_*c*_ on the edge of the blast-hole would be:

$$P_{c}=\rho_{m}\cdot c_{p}\cdot \nu_{c}$$ (3)

where *ρ*_*m*_ is the density of coal, kg/m^3^. *v*_*c*_ is the propagation speed of mass point, m/s, *v*_*c*_ = (*c*_*p*_ − *a*)/*b*. The experiment can obtain constant determined a, b.

The radius of the crushed area *r*_*c*_ is calculated by the formula (2) and (3) obtained above, shown in formula (4):

$$r_{c}=r_{b}\cdot {\left(\frac{b\cdot P_{m}}{\rho_{m}\cdot c_{p}\cdot (c_{p}-a)}\right)}^{\frac{1}{\alpha }}$$ (4)

The length of the closed area along the radial direction of blasting is:

$$L_{0}=r_{c}-r_{b}=r_{b}\cdot \left[{\left(\frac{b\cdot P_{m}}{\rho_{m}\cdot c_{p}\cdot (c_{p}-a)}\right)}^{\frac{1}{3}}-1\right]$$ (5)

When the shock wave attenuates, it will continue to propagate and decay in the form of a stress wave in the coal seam. Taking into account the Poisson effect, the attenuation of peak tensile stress of stress wave in the fracture zone is:

$$\sigma =P_{c}\cdot \frac{\mu }{1-\mu }\cdot {\left(\frac{L}{r_{c}}\right)}^{-\alpha }$$ (6)

where α is the stress wave attenuation coefficient, and *α* = 2 − *μ*/(1 − *μ*). μ is the Poisson’s ratio.

When the tangential stress peak of stress wave attenuates to below the tensile strength of coal body, the crack extension will end. By swapping σ for *R*_*t*_ in Eq (6), we can obtain the radius of radial fracture zone under the action of stress wave:

$$r_{t}=r_{c}\cdot {\left(\frac{P_{c}}{R_{t}}\cdot \frac{\mu }{1-\mu }\right)}^{\frac{1}{\alpha }}$$ (7)

The range of the crack propagation along the radial direction under the stress wave:

$$L_{1}=r_{t}-r_{b}$$ (8)

When the stress wave decays to the level that crack cannot be extended, the methane produced by the blasting will quickly penetrate the fracture channel created by the stress wave and continue to exert static pressure on the fracture. This reaction continues the fracture expansion. When the dissipated pressure is less than the fracture toughness of coal *K*_*Id*_, the crack does not continue to expand. The ultimate stress intensity *K*_*Id*_ when it no longer expands:

$$K_{Id}=\sqrt{\pi (L+r_{b})}\left[\left(1-\frac{2}{\pi }\right)P_{L}-\sigma \right]$$ (9)

where *L* is the crack final extend length, m; *P*_*L*_ is the stress wave when it no longer expands, the methane pressure generated by the blasting, *Pa*; and *σ* is the crustal stress, *Pa*.

The critical condition for the crack to stop expanding is:

$$\sqrt{\pi (L+r_{b})}\left[\left(1-\frac{2}{\pi }\right)P_{L}-\sigma \right]=K_{Id}$$ (10)

where *K*_*Id*_ is the dynamic fracture toughness, *MPa*·m^1/2^. *K*_*Id*_ = (1.6)*K*_*Ic*_, *K*_*Ic*_ is the Static fracture toughness, *MPa*·m^1/2^.

As the stress wave no longer expands outward, the pressure attenuation law of the methane produced by the explosion satisfies the relationship as follows:

$$P_{L}=P_{m0}\cdot {\left(\frac{r_{b}}{r_{b}+L}\right)}^{1.5}$$ (11)

where *P*_*m0*_ is the explosion pressure, *Pa*. The range where the fracture can eventually be extended is:

$$L=0.873\times \sqrt[{3}]{\frac{P_{m0}}{K_{Ic}}}\sqrt{r_{b}}-r_{b}$$ (12)

Therefore, we can conclude the relation formula *L*_*H*_ between blasthole and control-hole as follows:

$$L_{1}+r_{b}\leq L_{H}\leq L+r_{b}$$ (13)

According to actual production conditions of the Nanting coal mine, we chose emulsion explosions in the coal mine with a density of 1050~1300 kg/m^3^, detonation velocity of 3000~5000 m/s, and burst pressure of 10 GPa. The uncoupling charge coefficient k is 1.57, and the diameter between blasthole and control-hole is 94 mm. The extension range of the borehole fracture is between 3.11 and 5.24 m after blasting (blasthole spacing between 6.22 m and 10.48 m) by formula (12). Shown are the basic parameters (Table 1).

**Table 1:** Basic parameters.

Parameter (unit)	value	Parameter (unit)	value
Coal density (kg/m^3^)	1350	methane dynamic viscosity (Pa·s)	1.12×10^−5^
Modulus of elasticity of coal (Pa)	3×10^9^	Adsorption constant *a*(m^3^/t)	23.09
Average methane pressure (Pa)	0.3×10^6^	Adsorption constant *b*(MPa^-1^)	0.91
Initial porosity (%)	12.63	Average moisture of coal seam (%)	5.24
Poisson’s ratio of coal	0.29	Average ash content of coal seam (%)	7.17
Tensile strength (Pa)	3×10^9^	Static fracture toughness (MPa·m^1/2^)	0.47
Internal friction angle (°)	33	Cohesion (Pa)	1.3×10^6^
Drilling diameter (mm)	94	Distance between borehole and bottom plate (m)	1.2
Borehole inclination (°)	6~8	Drilling direction	Vertical coal wall

## 3 The numerical simulation of reasonable blast-hole spacing

### 3.1 Comparison of effective extraction radius before and after blasting

COMSOL is used here to simulate the borehole radial, and the effective extraction radius of coalbed methane before and after the blasting was simulated at a different permeability [17,18]. The model size is 10 m × 20 m and the negative pressure is 14 *kPa*. The basic parameters are given in Table 1.

#### 3.1.1 Effective extraction radius before blasting’s

**Fig 2:**
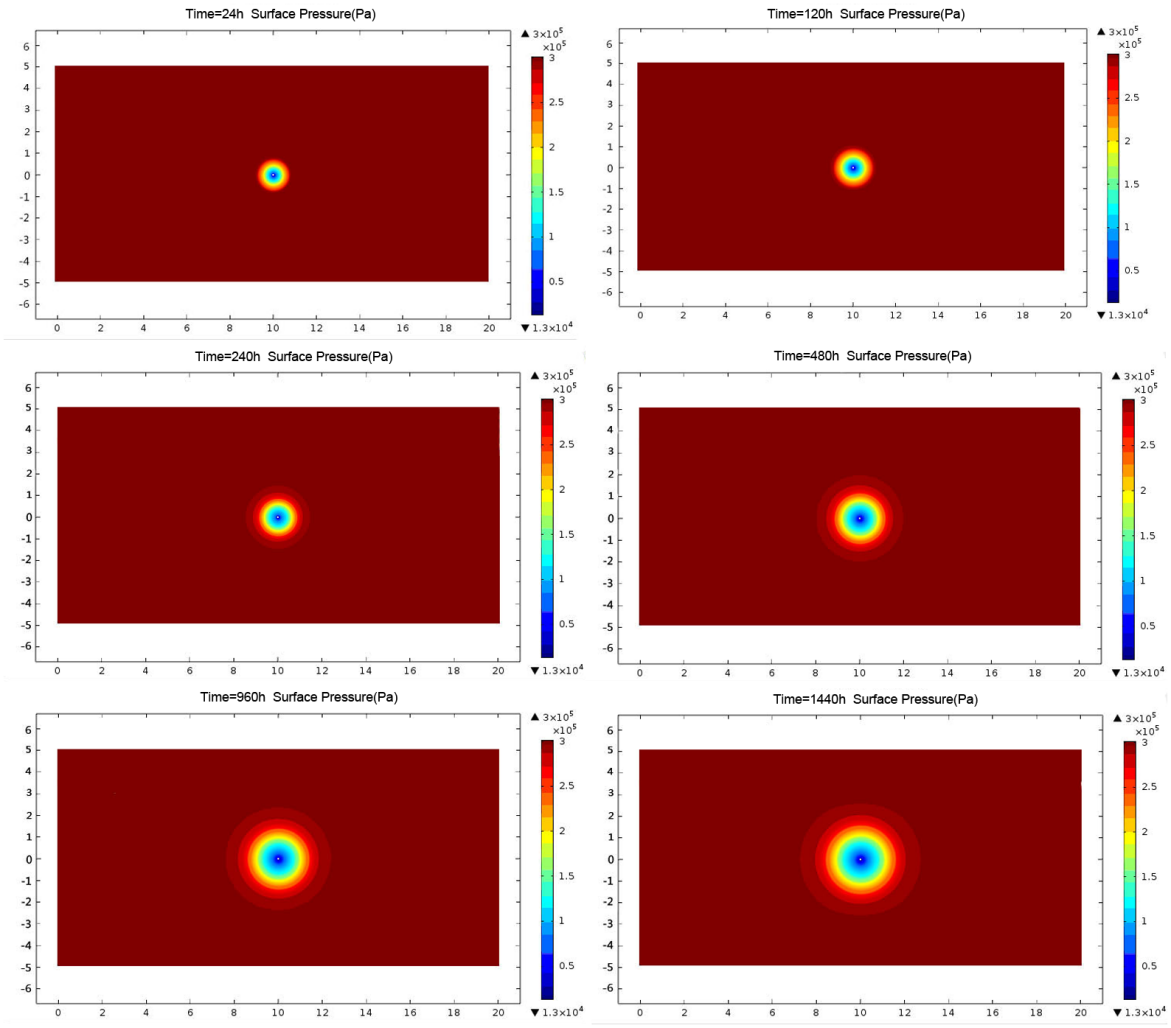
Cloud chart of borehole pressure before blasting.

**Fig 3:**
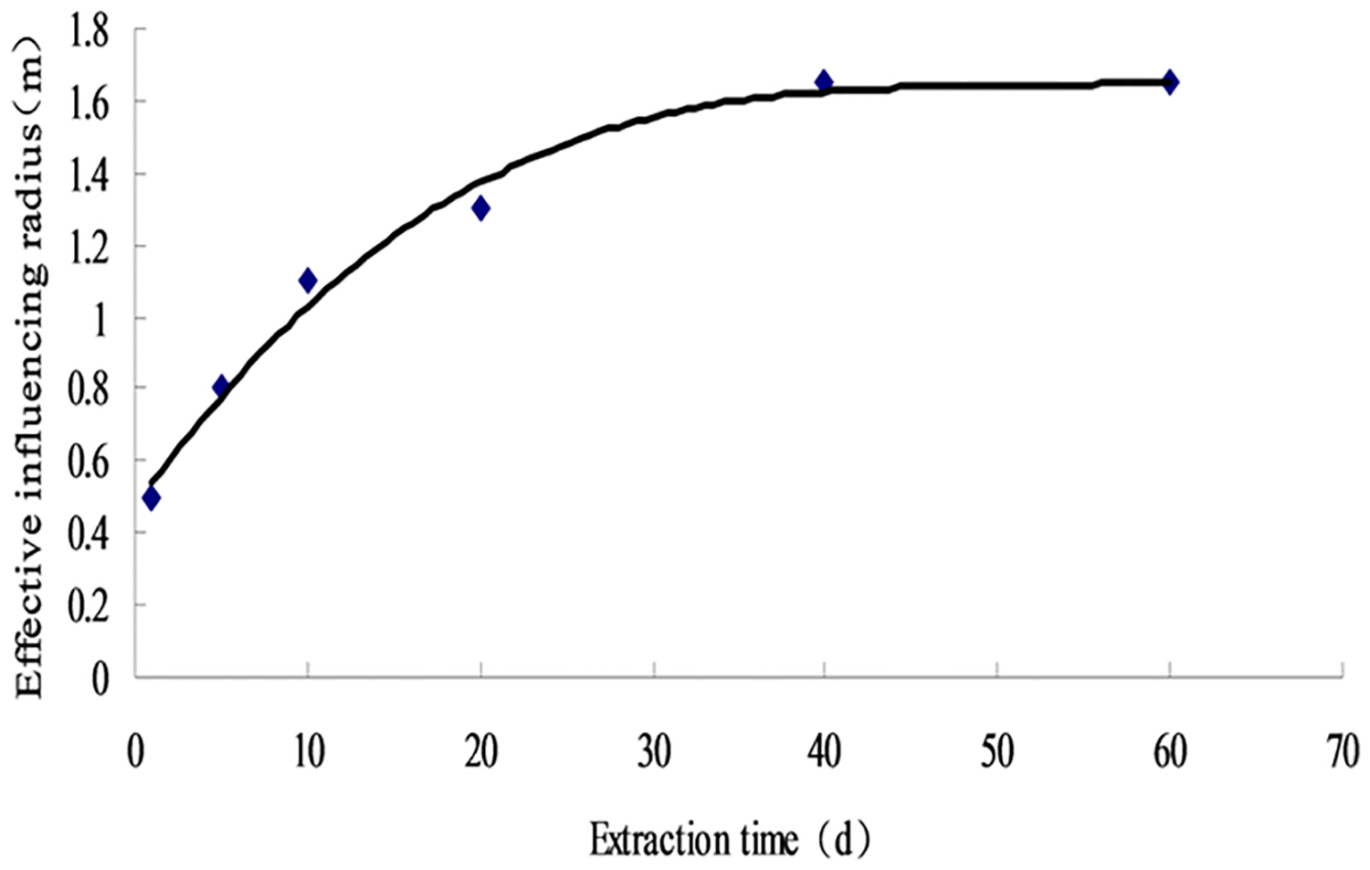
Fitting curve of pumping radius variation.

At certain extraction negative pressure, the stress nephogram of the borehole effective extraction radius varies with the extraction time before blasting was shown in Fig 2 The radius at different time points is calculated and fitted by function curve (S1 Table; Fig 3).

The effective extraction radius of the borehole during the entire extraction process shows three stages: increasing, slowly increasing, and unchanged (Figs 2 and 3). In the first 40 d, the radius exponentially increases to 1.6 m before extraction. It turns to the slowly increase stage from 40 d to 60 d, and slightly increases to 1.7 m at 60 d. In conclusion, the effective extraction radius of the borehole before blasting is 1.7 m.

#### 3.1.2 Effective extraction radius after blasting

**Fig 4:**
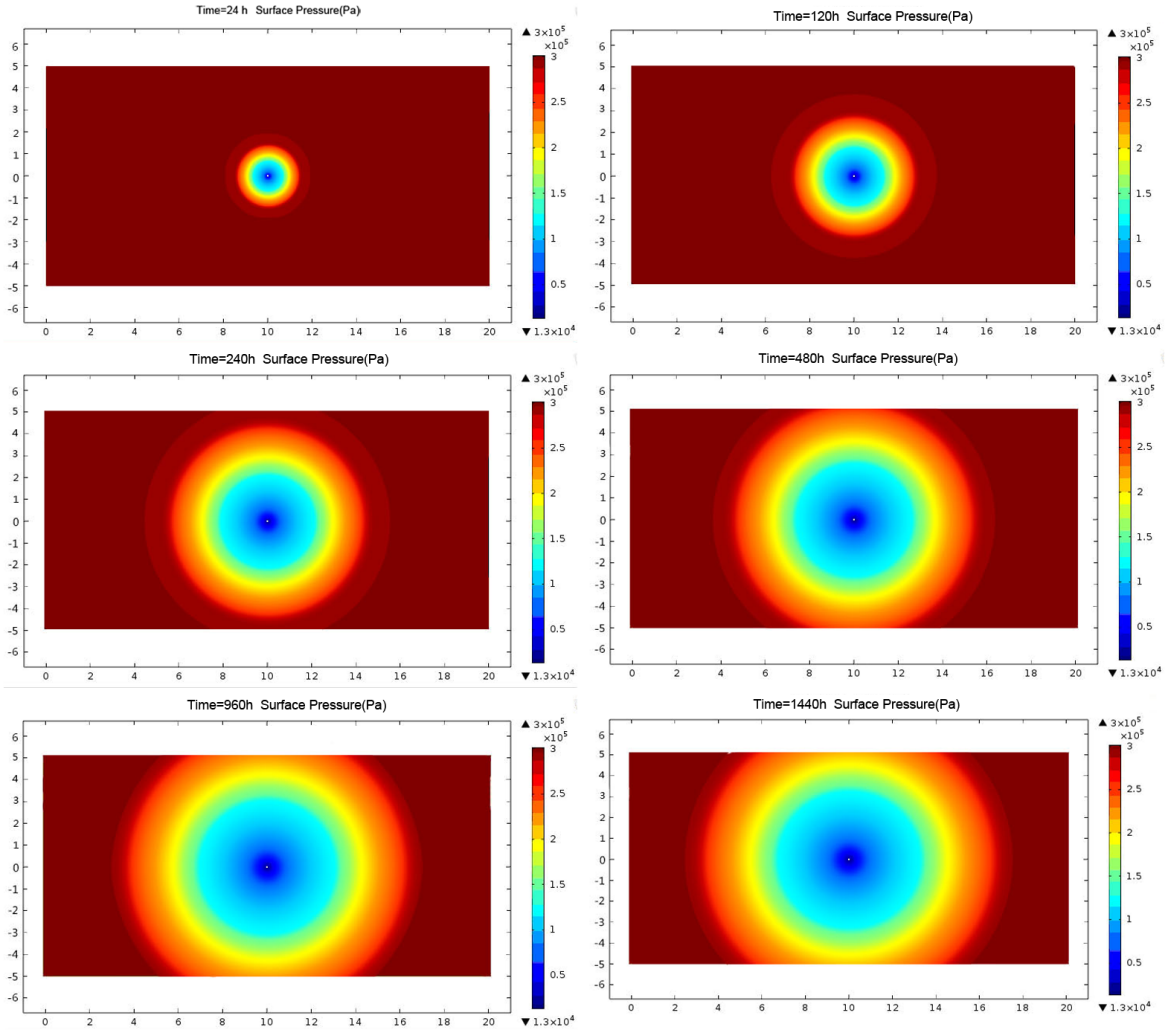
Cloud chart of borehole pressure after blasting.

**Fig 5:**
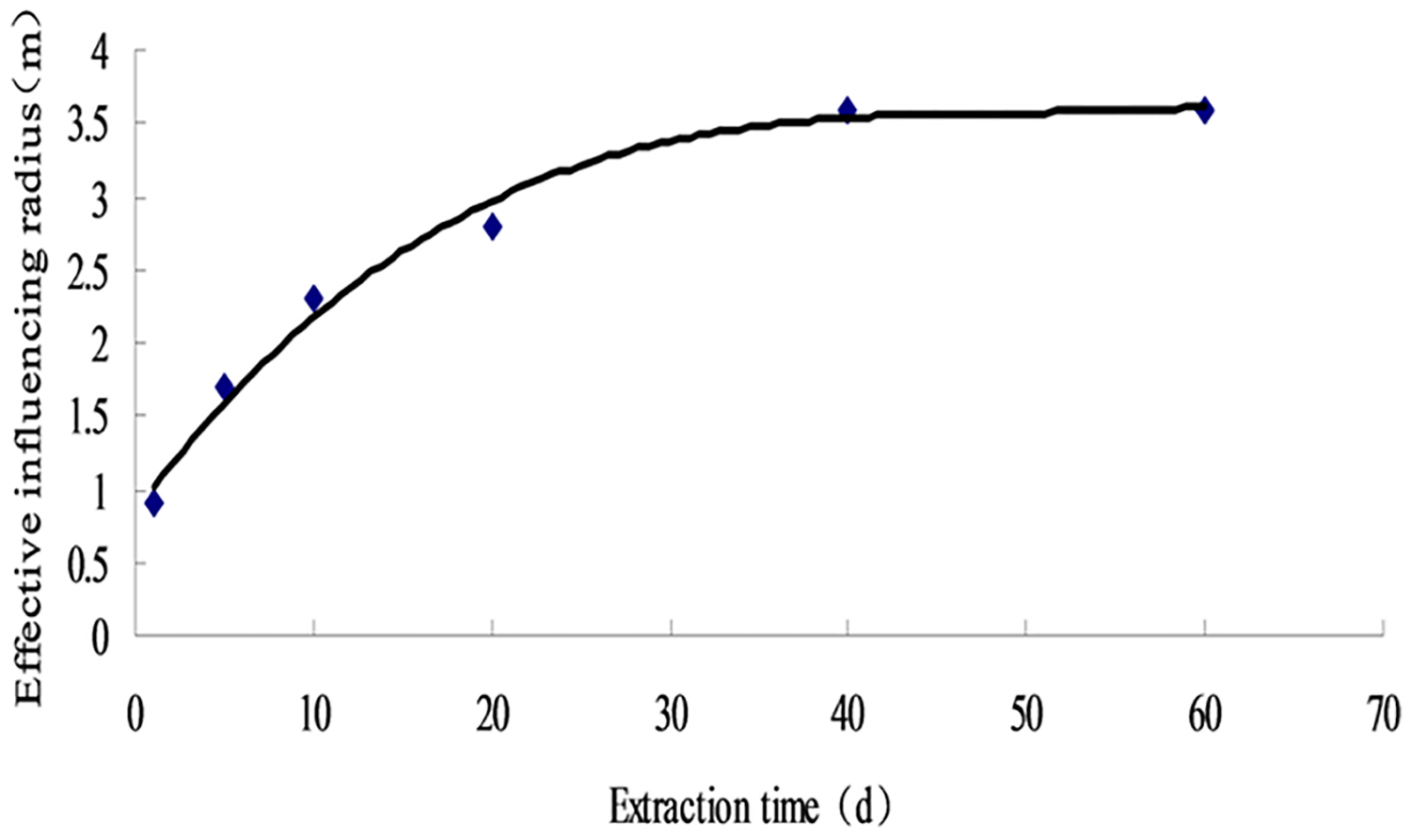
Fitting curve of variation of pumping radius.

After blasting, the cracks around the borehole have been greatly extended and penetrated, with a permeability coefficient increase to 2.58 m^2^/(MPa^2^·d). Under the condition of pumping negative pressure, the effective radius of the borehole can be calculated with the continuous stress nephogram (Fig 4). The radius of different time points is calculated, and the curve is fitted by function (S1 Table; Fig 5).

The growth rate of the extraction radius after blasting shows three stages: large growth rate, slow growth rate, and zero growth rate (the radius remains unchanged) (Fig 5). The effective radius of the first 40 d greatly increases, and it continues to increase to 3.5 m at 40 d. It changes to slow growth during 40 d and 60 d. It then becomes 3.6 m when the time has reached 60 d, where it ceases to increase. In conclusion, it stops increasing and remains steady.

Compared with the simulation results, the stress balance state of the coal seam is broken after blasting. Many cracks are produced owing to the instability of the coal seam, and the permeability of the coal seam is greatly raised. The effective radius of drilling is increased from 1.7 m to 3.6 m, and this positive effect is achieved by blasting. To avoid the blind area between two extraction holes, we use the method to rapidly decrease methane content in the coal seams and shorten the pre-pumping time. The cracks between the blasting holes are fully interconnected. Finally, the reasonable and effective extraction radius of the drilling hole is 3.5 m.

### 3.2 Crack propagation in pre-splitting blasting with different hole spacing

It is key to choose reasonable drilling spacing to eliminate blasting blank space and improving anti-reflection effect [19]. The pre-splitting blasting effects of blasting hole spacing of 10 m, 8 m, and 7 m were chosen and simulated by FLAC3D, while the blasting hole and control hole spacing were kept at 5 m, 4 m, and 3.5 m. The development of fractures after blasting was evaluated by observing the distribution of plastic zone between the blasting holes. The model size is 20 m × 70 m × 10 m, and the overburden stress is 10 *MPa*. The basic parameters are given in Table 1.

**Fig 6:**
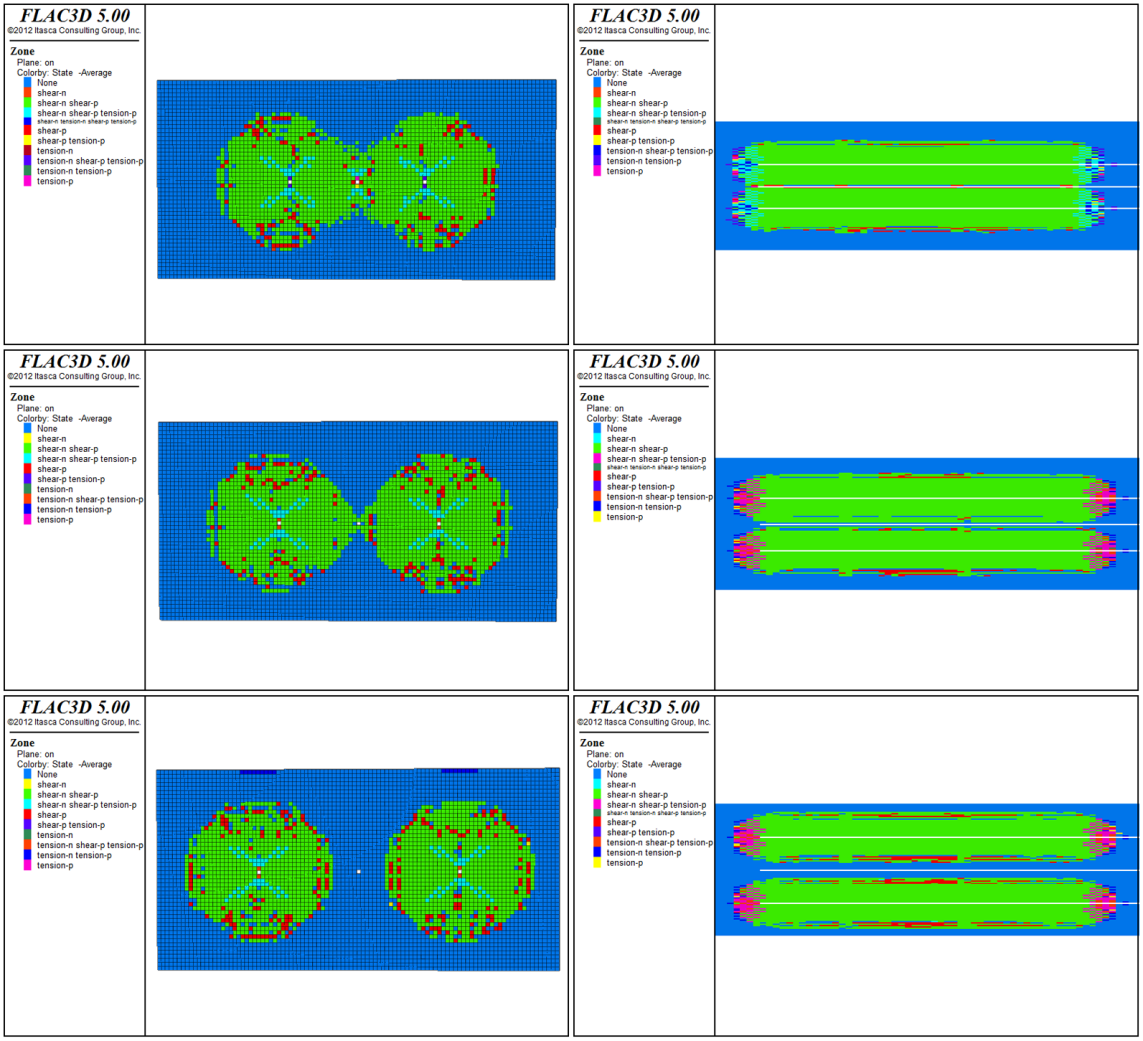
The plastic section and distribution of three different hole spacings.

Fig 6a shows that when the hole spacing is 10 m, a large area between the blasting holes is not affected by blasting, and the cracks between the control hole and the blasting hole are not penetrated. When the hole spacing is 8 m, the effective range of blasting increases more visibly than the 10 m case. In the control of hole direction, the crack penetrates part of the area. As given in Fig 6c, when the hole spacing is 7 m, the blank areas between the drilling holes are eliminated and the surrounding cracks can be fully interconnected.

Comparing the three simulation cases, when the distance between the two blasting holes is large, the control hole has no effect on the crack propagation. However, as the distance between the two blasting holes decreases, the effect of the control hole becomes increasingly important. As can be seen from Fig 6b and 6c, the adjacent blasting holes between the fissure development area are significantly greater than the blasting hole lateral development area.

## 4 Analysis of on-site measurement

In the field evaluation, four test parameters such as single hole methane extraction rate, methane extraction rate, coal permeability coefficient, and borehole limit methane extraction are chosen to analyze their effects on the blasting performance [20–23].

### 4.1 Test plan

The study was not carried out on private land. Specific permissions were required for these locations/activities. Site sampling site and test site were all from Tingnan coal mine. Approval person: Haibo Zhao.

I confirm that the field studies did not involve endangered or protected species.

**Fig 7:**
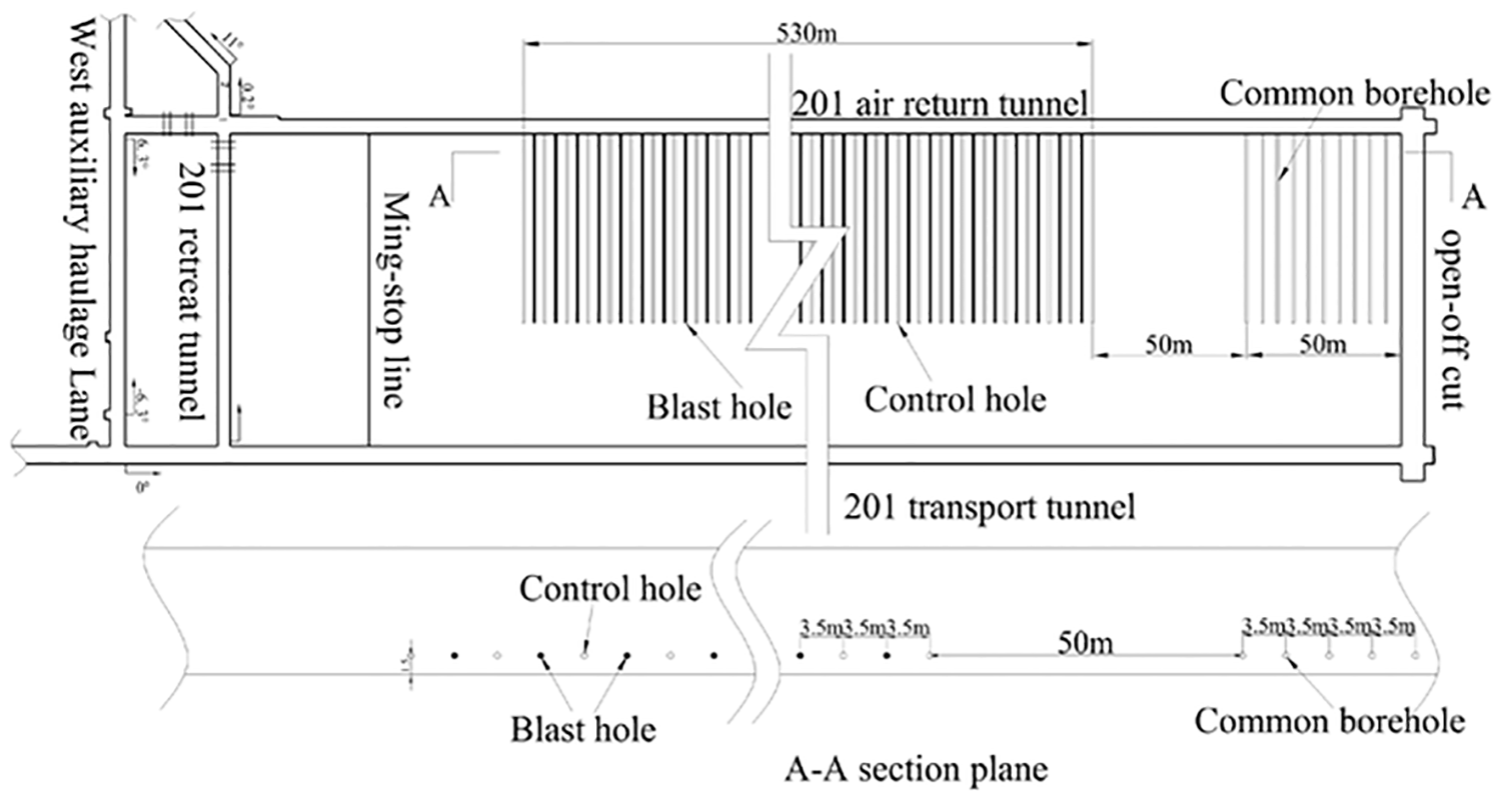
Experimental arrangement of borehole.

In order to reduce the influence of blasting vibration on the general mining area during the test and to enhance the contrast between the two cases, the working faces were divided into two sections: 14 ordinary bedding extraction holes and 151 pre-splitting blasting holes 100 m away from the working faces. The control hole was used as the extraction hole in the mining system for methane drainage. The drilling spacing was 3.5 m and the hole depth was 60 m, as shown in Fig 7.

### 4.2 Experiment results

#### 4.2.1 Analysis of borehole methane extraction

**Fig 8:**
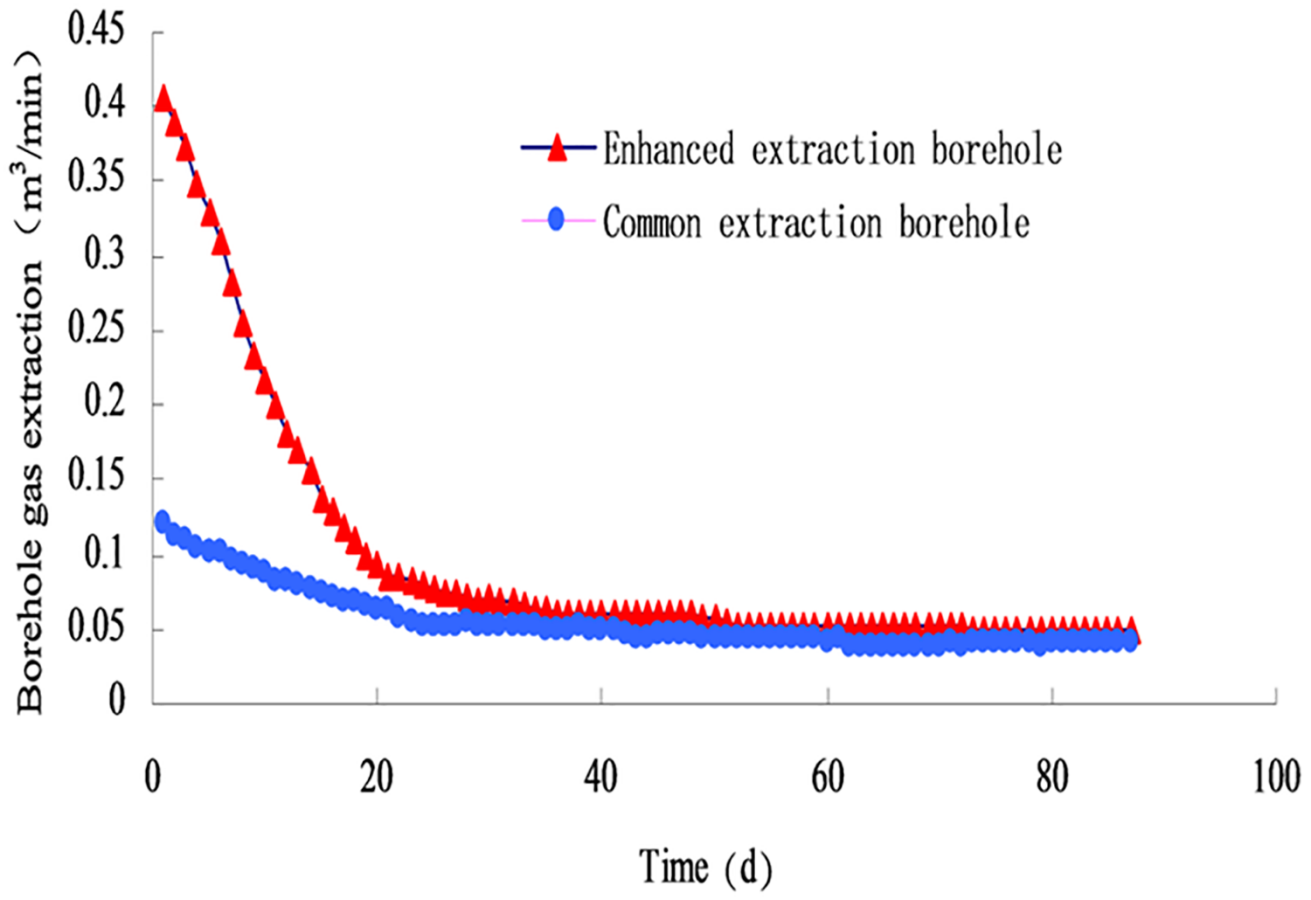
Variation curve of pumping amount with continuous mining.

The pumping capacity of the blasting and the ordinary section is measured by the orifice flow-meter, the high concentration methane detector, and the dry pipe automatic measuring device. The average pumping amount of all the boreholes is compared. The average pumping rate of the drilling is given in Fig 8 (S2 Table).

The slope of the methane extraction rate curve of the two borehole types under the same negative pressure quickly decreases in the first 15 d, showing that the methane flow rate at the initial stage of pumping is larger. However, the attenuation rate of the intensified pumping borehole is much larger than that of the ordinary borehole. From 15 d to 35 d, the curve of the extraction amount slope slowly decreases, and the pumping rate gradually decreases. After 35 d, the attenuation rate of the two boreholes is relatively slow, and finally the state is basically unchanged.

By means of statistical calculation, the average extraction volumes of enhanced drilling and conventional boreholes are 7910.9 m^3^ and 3347 m^3^, respectively. The extraction capacity of the enhanced drilling hole is 2.36 times the conventional borehole within the 3 months extraction. The effect of pumping is noticeably improved.

#### 4.2.2 Analysis of methane extraction rate

The comparison of the average extraction rate after blasting between the enhanced and normal pumping:

$$\eta =\frac{X_{0}-X_{c}}{X_{0}}\times 100\%$$ (14)

where *η* is the extraction rate, *X*_0_ is the original methane content of coal, m^3^/t. *X*_c_ is the methane content after coal extraction, m^3^/t.

The methane content of raw coal in 201 working surfaces was measured to be 6.64 m^3^/t. After 3 months of extraction, the coal was measured in the ordinary pre-pumping section and the blasting section; the methane content was 4.56 m^3^/t and 3.27 m^3^/t, respectively. Calculated by formula (14), the extraction rate of the common pre-pumping zone is 31.3% and the extraction rate after intensified pumping is 50.8%, which is 1.62 times as much as before. As can be observed, the effect of extraction is obviously improved.

#### 4.2.3 Permeability coefficient of coal seam

The comparison of the permeability coefficient of the coal seam before and after extraction is one of the most important indexes to evaluate the pumping efficiency. So far, it is difficult to directly measure the permeability of coal seams. The borehole methane extraction is used instead of the drilling flow, the permeability of the coal seam is indirectly converted. The results are compared and analyzed, and the extraction efficiency is evaluated. Based on the calculation principle of borehole radial unstable flow, the permeability coefficient of coal seam is calculated by using the fitted equation of permeability improvement in coal seam (Table 2).

**Table 2:** Optimization calculation equation revised.

Time criterion F_0_ = *B*λ	Constant *A*	Constant *B*	AB value range	Coefficient of permeability of coal seam λ
10^−2^~1	$A=\frac{qr_{1}}{P_{0}^{2}-P_{1}^{2}}$	$B=\frac{4tP_{0}^{1.5}}{\alpha r_{1}^{2}}$	<1	λ = A^1.61^B^0.61^
1~10			1~5.56	λ = A^1.34^B^0.34^
10~10^2^			5.56~35.43	λ = 1.19A^1.24^B^0.24^
10^2^~10^3^			35.43~256.64	λ = 1.58A^1.16^B^0.16^
10^3^~10^5^			256.64~16 233.63	λ = 2.11A^1.11^B^0.11^
10^5^~10^7^			>16 233.63	λ = 3.16A^1.07^B^0.07^

Where P_0_ is absolute methane pressure of coal seam, MPa. *P*_1_ is methane pressure in drilling hole, generally 0.1 MPa. *r*_1_ is drilling radius, m. λ is coefficient of permeability of coal seam, m^2^/(MPa^2^·d). *q* is methane flow per unit area of borehole coal wall at *t* time, m^3^/(m^2^·d). *Q* is total methane flow at boreholes at *t* time, m^3^·d^-1^. *L* is length of hole to see coal, m. α is methane content coefficient of coal seam, m^3^/(m^3^·MPa^0.5^).

**Fig 9:**
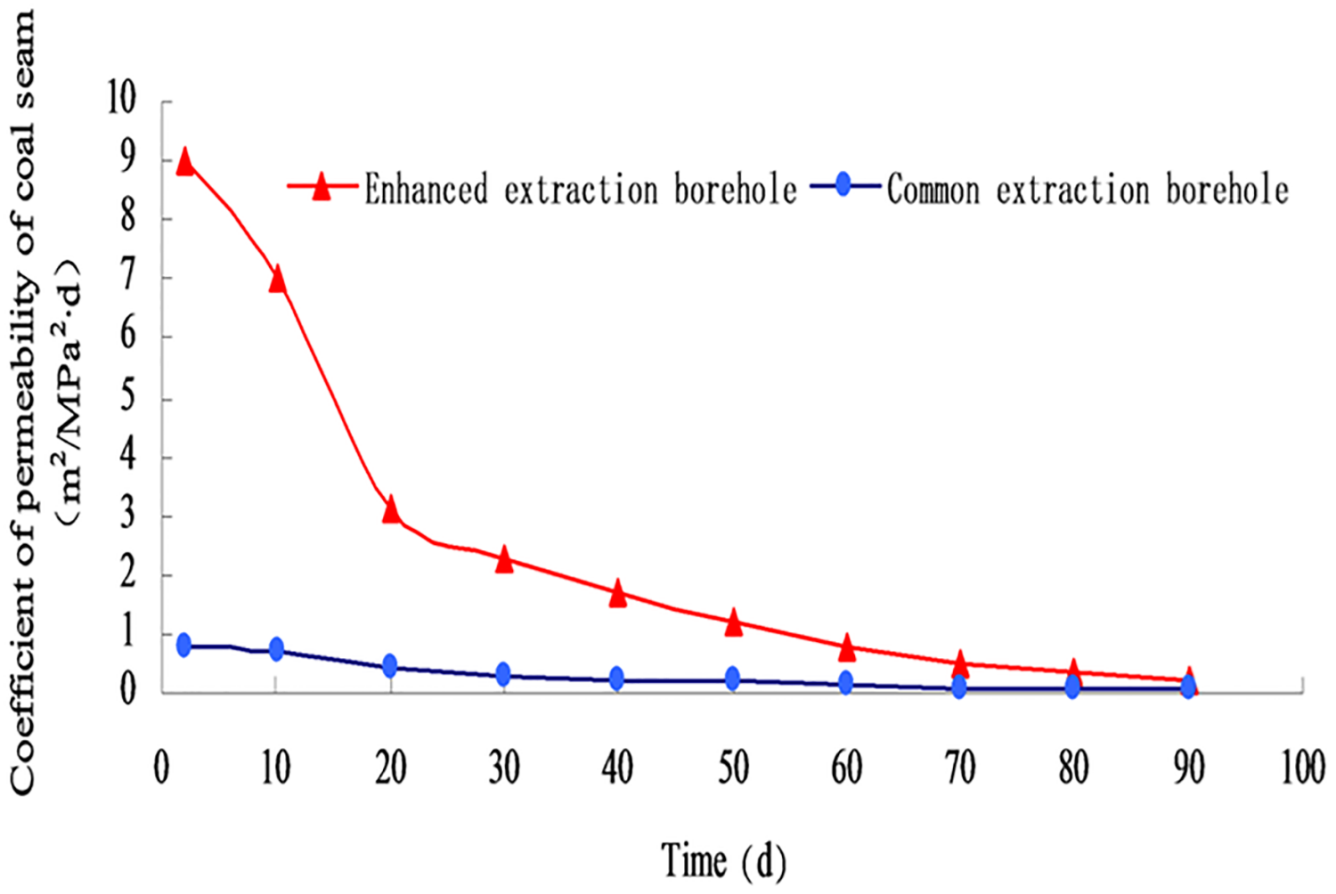
Variation curve of permeability coefficient of coal seam.

According to the above optimized equation, the average value of the permeability coefficient of the conventional mining area and the strengthened mining area is calculated under different extraction times, and the results are plotted, which is shown in Fig 9 (S3 Table; S4 Table).

It can be seen from Fig 9 that the permeability coefficient of the coal seam in the intensive pumping area is about 10 times that of the conventional extraction area, indicating that the bursting of the surrounding hole is expanded and the permeability coefficient of the coal seam expanded and increased. In the first 20 d, some fissures produced by the blasting were drastically decreased by the methane content, the stress concentration area was affected by pressure and other factors, and it gradually entered the compaction process. The permeability coefficient of the coal seam also showed different stages of decreasing, slowing down, and gradually stabilizing. After 90 d, the permeability coefficient of the coal seam was basically stable, but the permeability coefficient of the coal seam was three times higher than that of the ordinary. This denotes that some of the fissure space generated during the blasting is irreversible.

#### 4.2.4 Analysis of drilling limit methane extraction

At the same coal seam, with the same extraction system and extraction parameters, the effect of extraction is compared. The limited methane extraction is also one of the important indexes to evaluate the effect of drilling methane drainage. As the methane extraction is always in operation, it is important to compare the extraction enhancement. we used extraction quantity instead of drilling methane natural emission operation. Thus, the limited amount of drilling methane can be calculated.

According to the real-time data measured by the two pumping methods, the methane emissions at different time points were analyzed, and the regression analysis was carried out by multivariate statistical analysis. Thus, we can obtain the equation of natural methane emission:

$$q_{\text{t}}=q_{0}\cdot e^{-\alpha t}$$ (15)

where *q*_t_ is natural methane emission at *t* time, m^3^·min^-1^; *q*_0_ is natural methane emission from initial boreholes, m^3^·min^-1^; α is attenuation coefficient, d^-1^; and t is self-drainage time of drilling hole, d.

**Fig 10:**
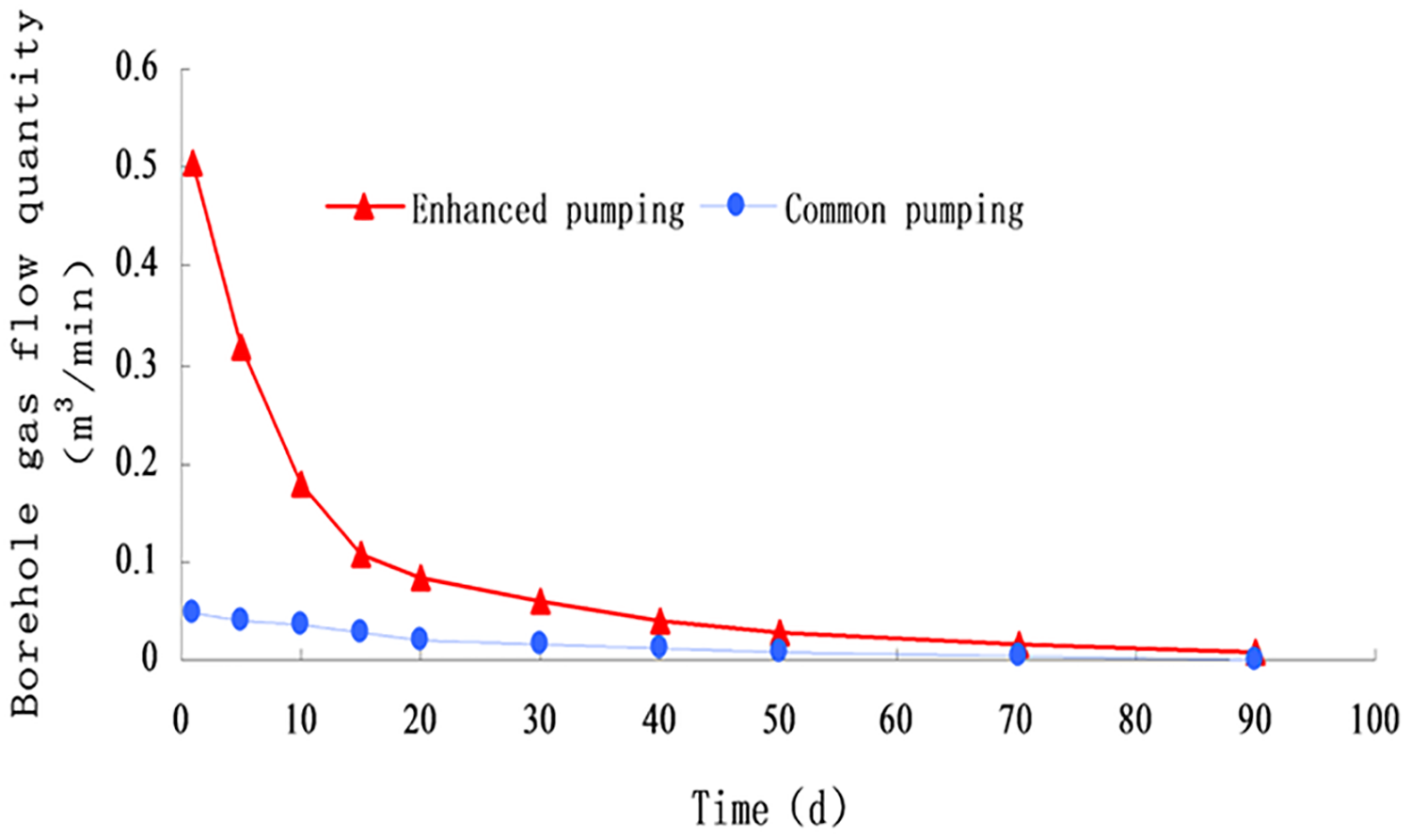
Variation curves of methane flow in boreholes.

Based on the measured data obtained from on-site sampling, the extraction amount of the two drainage types were obtained, as shown in Fig 10 (S5 Table).

**Fig 11:**
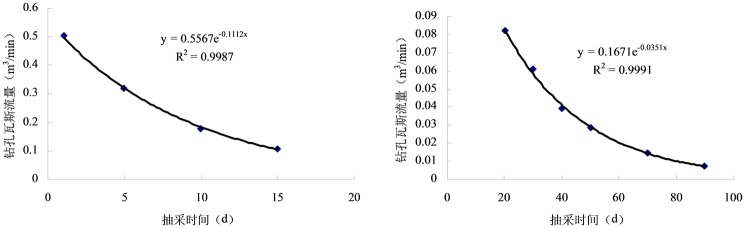
Regression curve of methane flow for intensified extraction.

**Fig 12:**
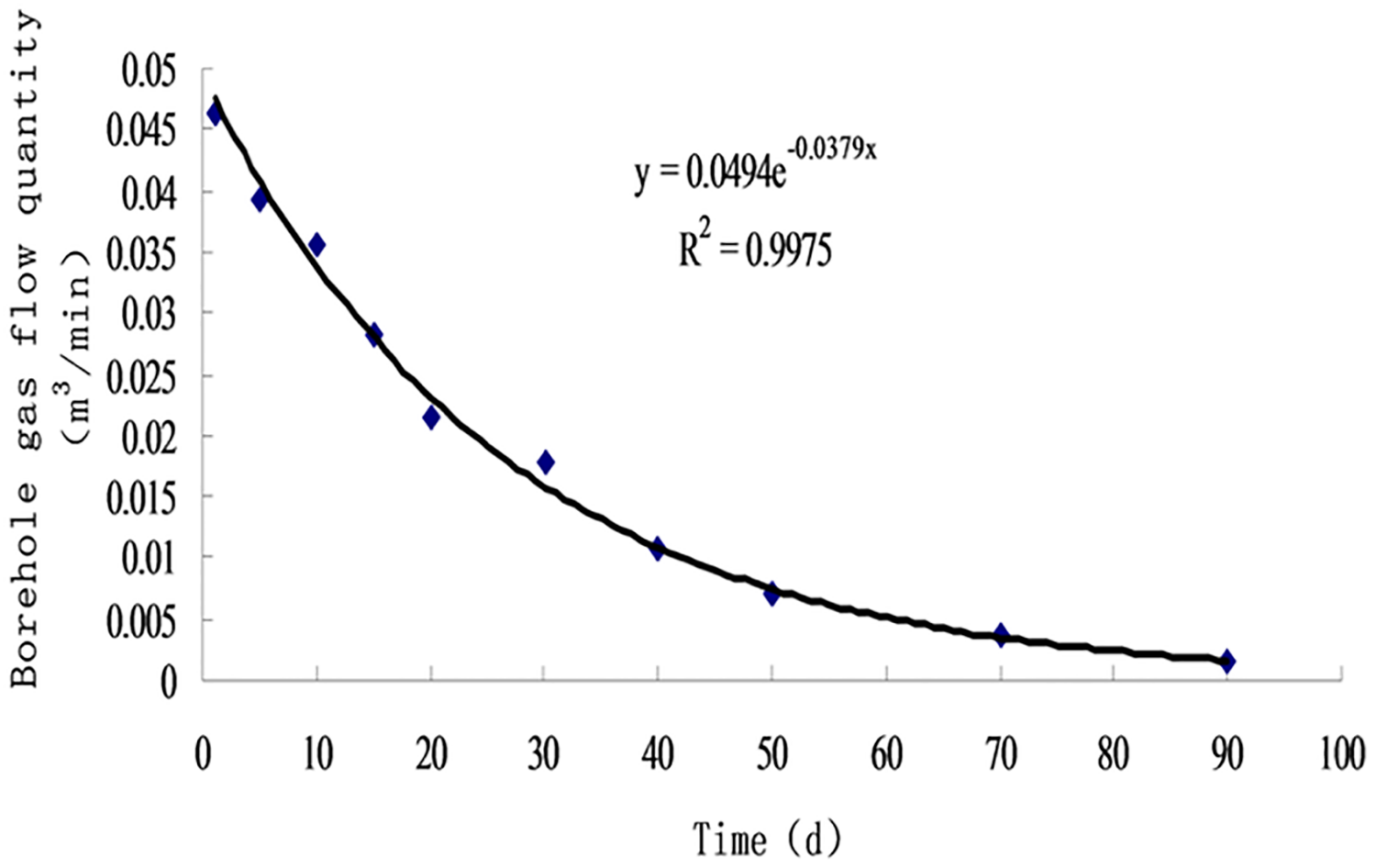
Regression curves of methane flow in common extraction boreholes.

From the numerical calculation of the nodes in Fig 10, it can be concluded that the methane flow rate of the enhanced boreholes at the beginning of the extraction is 5.1 times that of the conventional drilling, and the decay rate of the first 15 d is larger than that after 15 d with a large difference in attenuation. To reflect the change law of methane flow in a more detailed and accurate way, the intensive drainage of the first 15 d and after 15 d will be carried out, and the general sampling data will be processed as a whole, as shown in Figs 11 and 12.

As can be seen from Fig 11, the regression coefficient of the fitting curve of methane flow after drilling is 99.8% and 99.9%. The regression equation is:

$$\{\begin{array}{ll} q_{\text{t}1}=0.5567e^{-0.1112t}, & t\leq 15\text{d}\\ q_{\text{t}2}=0.1671e^{-0.0351t}, & t>15\text{d} \end{array}$$ (16)

From Fig 12, the regression equation of methane flow in common extraction boreholes can be obtained:

$$q_{\text{t}3}=0.0494e^{-0.0379t}$$ (17)

The total emission amount of methane at any moment by formula (15) is obtained, and the total emission amount of *t* at any time Q_t_ is obtained:

$$Q_{\text{t}}=1440\int_{0}^{t}q_{t}dt=1440\int_{0}^{t}q_{0}e^{-\alpha t}dt=\frac{1440q_{0}\left(1-e^{-\alpha t}\right)}{\alpha }=Q_{\text{j}}\left(1-e^{-\alpha t}\right)$$ (18)

where *Q*_t_ is total natural methane emission during drilling at any time *t*, m^3^; and *Q*_j_ is borehole limited methane emission, m^3^.

According to the regression equation obtained above, the maximum extraction methane amount of the enhanced extraction borehole and conventional borehole is 9898.52 m^3^ and 1876.94 m^3^, respectively. After the enhancement, the drilling limited methane extraction is 5.27 times the conventional borehole, and the pumping effect is better than the conventional drilling.

## 5 Conclusions

Through the analysis of the mechanisms of pre-crack blasting fracture expansion, a mechanical model for deep hole pre-splitting blasting crack expansion was established. Combined with on-site basic data of Tingnan Coal Mine, it is calculated that the single drilled fracture crack extension range is 3.11~5.24 m, which provides an theoretical basis for selecting the appropriate hole spacing.

Using COMSOL simulation software to simulate the effective gas drainage radius of a coal seam from a two-dimensional perspective on a single borehole radial, the least squares fitting method was used to analyze the simulated data, and obtained the effective extraction radius of the borehole before blasting is 1.7 m, the effective drilling extraction radius after pre-split blasting in a deep hole is 3.6 m. The results of comparative analysis show that the stress equilibrium state of coal seam is broken after blasting. The coal seam is unstable and produces a lot of cracks, which greatly improves the permeability of coal seam to obtain good blasting effect. After pre-split blasting in a deep hole, the effective drilling extraction radius of single hole is 3.6 m, which is within the range of theoretical calculations and meets the technical requirements.

In order to eradicate white zones in the blasting space, the surrounding cracks can be fully penetrated throughout the surroundings. FLAC3D numerical simulation software was used to obtain accurate and scientific calculations. After simulating the distribution of plastic zone between two blast holes at different intervals from a three-dimensional angle, and evaluating the situation of crack development between blasting holes after blasting, it is concluded that two blasting holes in the middle and sides of the gap is the best situation for crack development when the hole spacing is 7 m. Therefore, the optimal blasting hole spacing is determined to be 7 m.

## Supporting information

S1 Table Using COMSOL to simulate before and after blasting, effectively influencing radius over time. (DOC)

S2 Table Methane extraction amount from normal over time borehole and pre-split blasting borehole. (DOC)

S3 Table The permeability coefficient of coal seam of pre-split blasting area. (DOC)

S4 Table The permeability coefficient of coal seam of conventional area. (DOC)

S5 Table Borehole methane flow monitoring data. (DOC)
