# Supplementary material for: Permeability enhancement of deep hole pre-splitting blasting in the low permeability coal seam of the Nanting coal mine
Source: PLoS One. 2018 Jun 28;13(6):e0199835. doi: 10.1371/journal.pone.0199835 (PMC6023211; doi:10.1371/journal.pone.0199835)
Supplement: S1 Table — (DOC) [file pone.0199835.s001.doc]

**S1 Table.** Using COMSOL to simulate before and after blasting, effectively influencing radius over time

| Extraction time/d | Effective extraction radius before blasting /m | Effective extraction radius after blasting /m |
| --- | --- | --- |
| 1 | 0.5 | 0.9 |
| 5 | 0.8 | 1.7 |
| 10 | 1.1 | 2.3 |
| 20 | 1.3 | 2.8 |
| 40 | 1.7 | 3.6 |
| 60 | 1.7 | 3.6 |
