# Supplementary material for: Permeability enhancement of deep hole pre-splitting blasting in the low permeability coal seam of the Nanting coal mine
Source: PLoS One. 2018 Jun 28;13(6):e0199835. doi: 10.1371/journal.pone.0199835 (PMC6023211; doi:10.1371/journal.pone.0199835)
Supplement: S2 Table — (DOC) [file pone.0199835.s002.doc]

**S1 Table.** Methane extraction amount from normal over time borehole and pre-split blasting borehole

| Extraction time /d | Methane extraction amount from normal borehole（m^3^/min） | Methane extraction amount from pre-split blasting borehole（m^3^/min） |
| --- | --- | --- |
| 1 | 0.12051 | 0.40743 |
| 2 | 0.11250 | 0.38919 |
| 3 | 0.10946 | 0.37247 |
| 4 | 0.10490 | 0.34966 |
| 5 | 0.10169 | 0.32838 |
| 6 | 0.10034 | 0.31014 |
| 7 | 0.09679 | 0.28277 |
| 8 | 0.09426 | 0.25541 |
| 9 | 0.08970 | 0.23412 |
| 10 | 0.08818 | 0.21588 |
| 11 | 0.08361 | 0.19916 |
| 12 | 0.08209 | 0.18243 |
| 13 | 0.08057 | 0.16875 |
| 14 | 0.07753 | 0.15507 |
| 15 | 0.07449 | 0.13839 |
| 16 | 0.07145 | 0.12858 |
| 17 | 0.06976 | 0.11838 |
| 18 | 0.06841 | 0.11082 |
| 19 | 0.06588 | 0.09970 |
| 20 | 0.06385 | 0.09305 |
| 21 | 0.06233 | 0.08605 |
| 22 | 0.05659 | 0.08401 |
| 23 | 0.05473 | 0.08201 |
| 24 | 0.05321 | 0.07849 |
| 25 | 0.05321 | 0.07697 |
| 26 | 0.05321 | 0.07493 |
| 27 | 0.05321 | 0.07297 |
| 28 | 0.05473 | 0.07264 |
| 29 | 0.05270 | 0.06993 |
| 30 | 0.05169 | 0.07010 |
| 31 | 0.05236 | 0.06993 |
| 32 | 0.05169 | 0.06926 |
| 33 | 0.05186 | 0.06537 |
| 34 | 0.05169 | 0.06385 |
| 35 | 0.05017 | 0.06233 |
| 36 | 0.05017 | 0.06081 |
| 37 | 0.05017 | 0.06081 |
| 38 | 0.05169 | 0.06081 |
| 39 | 0.05017 | 0.06081 |
| 40 | 0.04814 | 0.06064 |
| 41 | 0.04865 | 0.06041 |
| 42 | 0.04595 | 0.06021 |
| 43 | 0.04409 | 0.06001 |
| 44 | 0.04476 | 0.05981 |
| 45 | 0.04561 | 0.05964 |
| 46 | 0.04561 | 0.05941 |
| 47 | 0.04561 | 0.05927 |
| 48 | 0.04561 | 0.05901 |
| 49 | 0.04409 | 0.05852 |
| 50 | 0.04409 | 0.05801 |
| 51 | 0.04409 | 0.05603 |
| 52 | 0.04409 | 0.05270 |
| 53 | 0.04409 | 0.05220 |
| 54 | 0.04409 | 0.05253 |
| 55 | 0.04257 | 0.05203 |
| 56 | 0.04409 | 0.05269 |
| 57 | 0.04257 | 0.05286 |
| 58 | 0.04257 | 0.05303 |
| 59 | 0.04257 | 0.05218 |
| 60 | 0.04105 | 0.05235 |
| 61 | 0.04257 | 0.05218 |
| 62 | 0.03953 | 0.05218 |
| 63 | 0.03953 | 0.05201 |
| 64 | 0.03953 | 0.05235 |
| 65 | 0.03801 | 0.05151 |
| 66 | 0.03953 | 0.05201 |
| 67 | 0.03953 | 0.05235 |
| 68 | 0.03801 | 0.05252 |
| 69 | 0.03953 | 0.05201 |
| 70 | 0.03953 | 0.05235 |
| 71 | 0.04054 | 0.05235 |
| 72 | 0.03953 | 0.05117 |
| 73 | 0.04088 | 0.04965 |
| 74 | 0.04105 | 0.04965 |
| 75 | 0.04105 | 0.04965 |
| 76 | 0.04105 | 0.04999 |
| 77 | 0.04105 | 0.04948 |
| 78 | 0.04105 | 0.04914 |
| 79 | 0.03953 | 0.04965 |
| 80 | 0.04105 | 0.04982 |
| 81 | 0.04105 | 0.04982 |
| 82 | 0.04105 | 0.04965 |
| 83 | 0.04105 | 0.04965 |
| 84 | 0.04105 | 0.04965 |
| 85 | 0.04105 | 0.04982 |
| 86 | 0.04105 | 0.04999 |
| 87 | 0.04105 | 0.04999 |
| 88 | 0.04105 | 0.04999 |
| 89 | 0.04105 | 0.04999 |
| 90 | 0.04105 | 0.04999 |
