# Supplementary material for: Permeability enhancement of deep hole pre-splitting blasting in the low permeability coal seam of the Nanting coal mine
Source: PLoS One. 2018 Jun 28;13(6):e0199835. doi: 10.1371/journal.pone.0199835 (PMC6023211; doi:10.1371/journal.pone.0199835)
Supplement: S3 Table — (DOC) [file pone.0199835.s003.doc]

**S3 Table.** The permeability coefficient of coal seam of pre-split blasting area

| Time（d） | 2 | 10 | 20 | 30 | 40 | 50 | 60 | 70 | 80 | 90 |
| --- | --- | --- | --- | --- | --- | --- | --- | --- | --- | --- |
| No.6 borehole | 9.49 | 7.34 | 2.98 | 2.2 | 2.01 | 1.37 | 0.71 | 0.56 | 0.45 | 0.16 |
| No.8 borehole | 8.50 | 6.46 | 3.23 | 2.28 | 1.38 | 1.05 | 0.89 | 0.40 | 0.27 | 0.24 |
| Mean value | 9.00 | 6.90 | 3.10 | 2.24 | 1.69 | 1.21 | 0.80 | 0.48 | 0.36 | 0.20 |
