# Supplementary material for: Permeability enhancement of deep hole pre-splitting blasting in the low permeability coal seam of the Nanting coal mine
Source: PLoS One. 2018 Jun 28;13(6):e0199835. doi: 10.1371/journal.pone.0199835 (PMC6023211; doi:10.1371/journal.pone.0199835)
Supplement: S4 Table — (DOC) [file pone.0199835.s004.doc]

**S4 Table.** The permeability coefficient of coal seam of conventional area

| Time（d） | 2 | 10 | 20 | 30 | 40 | 50 | 60 | 70 | 80 | 90 |
| --- | --- | --- | --- | --- | --- | --- | --- | --- | --- | --- |
| No.9 borehole | 0.876 | 0.819 | 0.463 | 0.395 | 0.321 | 0.261 | 0.177 | 0.064 | 0.064 | 0.064 |
| No.10 borehole | 0.816 | 0.707 | 0.330 | 0.211 | 0.163 | 0.103 | 0.093 | 0.059 | 0.059 | 0.049 |
| Mean value | 0.846 | 0.763 | 0.396 | 0.303 | 0.242 | 0.182 | 0.135 | 0.061 | 0.061 | 0.061 |
