# Supplementary material for: Permeability enhancement of deep hole pre-splitting blasting in the low permeability coal seam of the Nanting coal mine
Source: PLoS One. 2018 Jun 28;13(6):e0199835. doi: 10.1371/journal.pone.0199835 (PMC6023211; doi:10.1371/journal.pone.0199835)
Supplement: S5 Table — (DOC) [file pone.0199835.s005.doc]

**S5 Table.** Borehole methane flow monitoring data

| Extraction time /d | Methane flow of boreholes in pre-split blasting（m^3^/min） | Methane flow of conventional boreholes（m^3^/min） |
| --- | --- | --- |
| 1 | 0.503571 | 0.046429 |
| 5 | 0.317857 | 0.039286 |
| 10 | 0.178571 | 0.035714 |
| 15 | 0.107143 | 0.028175 |
| 20 | 0.082143 | 0.021429 |
| 30 | 0.060714 | 0.017857 |
| 40 | 0.039286 | 0.010714 |
| 50 | 0.028571 | 0.007143 |
| 70 | 0.014286 | 0.003571 |
| 90 | 0.007143 | 0.001587 |
